# The Coexistence of Coping Resources and Specific Coping Styles in Stress: Evidence from Full Information Item Bifactor Analysis

**DOI:** 10.1371/journal.pone.0096451

**Published:** 2014-05-01

**Authors:** Jiaxi Zhang, Meng Cui, Wei Wang, Huijie Lu, Qing Wu, Xia Zhu, Danmin Miao, Yan Zhang, Xi Feng, Wei Xiao

**Affiliations:** 1 Department of Psychology, Fourth Military Medical University, Xi’an, China; 2 Xi’an Mental Health Center, Xi’an, China; 3 Foreign Language Teaching and Researching Office of Basic Education Department, Chongqing Communication Institute, Chongqing, China; University of Rochester, United States of America

## Abstract

**Background:**

Knowledge of coping styles is useful in clinical diagnosis and suggesting specific therapeutic interventions. However, the latent structures and relationships between different aspects of coping styles have not been fully clarified. A full information item bifactor model will be beneficial to future research.

**Objective:**

One goal of this study is identification of the best fit statistical model of coping styles. A second goal is entails extended analyses of latent relationships among different coping styles. In general, such research should offer greater understanding of the mechanisms of coping styles and provide insights into coping with stress.

**Methods:**

Coping Styles Questionnaire (CSQ) and Generalized Self-Efficacy Scale (GSES) were administrated to officers suffering from military stress. Confirmatory Factor Analyses was performed to indentify the best fit model. A hierarchical item response model (bifactor model) was adopted to analyze the data. Additionally, correlations among coping styles and self-efficacy were compared using both original and bifactor models.

**Results:**

Results showed a bifactor model best fit the data. Item loadings on general and specific factors varied among different coping styles. All items loaded significantly on the general factor, and most items also had moderate to large loadings on specific factors. The correlation between coping styles and self-efficacy and the correlation among different coping styles changed significantly after extracting the general factor of coping stress using bifactor analysis. This was seen in changes from positive (r = 0.714, p<0.01) correlation to negative (r = −0.335, p<0.01) and also from negative (r = −0.296, p<0.01) to positive (r = 0.331, p<0.01).

**Conclusion:**

Our results reveal that coping styles have a bifactor structure. They also provide direct evidence of coexisting coping resources and styles. This further clarifies that dimensions of coping styles should include coping resources and specific coping styles. This finding has implications for measurement of coping mechanisms, health maintenance, and stress reduction.

## Introduction

The development of positive psychology has brought a remarkable increase in attention to quality of life (QOL). This is despite the fact that individuals are exposed to various psychosocial stressors in all phases of their lives. Research has found that coping styles, personalities, and stress levels are significantly associated with QOL [1]–[4]. Certainly, stress is negatively associated with QOL, and the link between adverse or stressful life events and psychological and physical health has been firmly established since original proposals connecting the concept of coping with stress developed by Lazarus and colleagues in the early 1980s [5]–[9]. Recent evidence also shows links between stress and substance abuse [10], [11]. Substance abuse as a method of coping with stress has been of particular interest to researchers due to the unique association of stress with problematic substance use and with the development of related disorders [12]. In the field of psychosomatic medicine, studies also find that psychological stress can up-regulate inflammatory processes and increase disease risk [13]. Consequently, one conclusion is that stressful life events precipitate ill-health and psychological dysfunction. However, the degree of influence that these types of adverse events have on psychological and physical health, or the incidence of substance abuse remains a matter of debate. Some researchers have reported only a small effect of life stress on psychological and physical health, while others have found a significant effect [14].

One factor that may contribute to inconsistent results across studies involves the fact that people exhibit different levels of resilience in response to life stresses [15], such as coping styles and cognitive hardiness. Negative feelings that accompany stress often motivate the use of behavioral and cognitive strategies to cope with a difficult situation and to restore satisfaction to QOL [7]. Effective coping strategies to deal with stress can include successful engagement with adversity, which ‘steels’ individuals rather than sensitizing them. Studies found that differences in how individuals cope may have implications for health in the context of stress [16], [17]. Kobasa [18] also found that higher levels of stress are associated with increased opportunities for resilience in particular participants. Other researchers have argued the need to differentiate between protective factors, which are likely to be a given in an individual’s life, and protective mechanisms (such as coping style or explanatory style) which may be developed over the course of events in a person’s life [9], [11], [19]. Findings suggest that protective factors for stress may include coping (e.g., seeking social support) [20]. Thus, coping styles have been found to protect against the effects of stress and to attenuate its effects on psychological problems. Researchers in the field of psychosomatic medicine have shown that coping styles for addressing stress are related to the way in which persons with chronic illnesses deal with disease, pain, and adjustment [21], [22]. However, when considering various coping styles used across different situations, resulting conclusions are inconsistent. The protective effect of so-called adaptive coping styles, such as help seeking, may not have a significant effect; in fact, so-called maladaptive immature coping (e.g. avoiding coping) may become a protective factor in stress [9], [23], [24]. Additionally, protective mechanisms used with different coping styles and the latent relationships among them have not yet been clarified. Given the important role that coping styles play in linking stress and physical and psychological health, it is important to investigate underlying mechanisms of coping styles to develop knowledge that can be used to create relevant intervention strategies in stress.

A coping style reflects an individual's cognitive and behavioral efforts to change certain behaviors with the goal of dealing with specific internal and external environmental demands that are appraised as taxing or exceeding the individual's own resources [25]. The core aim of coping is “change” [6]. A state of well being can be achieved through “change” of external or internal conditions or by avoiding emotionally negative conditions and maintaining positive psychological states [26]. Although stress may be addressed by various methods, such as intentional, conscious, and goal-directed stress-management etc., these “changes” still need to be taken [6]. Hundreds of coping styles can be found in the literature on stress research. However, there remains no consensus on classification of these styles into a broader architecture. Common distinctions are often made between various contrasting coping styles, such as problem-focused and emotion-focused, approach and avoidant, and cognitive and behavioral [27]. Recently a number of researchers have tended to classify coping styles into immature and mature coping styles [28]. Immature coping styles include those of withdrawal, fantasy, self-reproach, projection, passive aggression, and acting out. Mature coping styles may include help-seeking, justification, problem solving, suppression, mature humor, and anticipation. Vaillant [29] expanded this classification method and proposed that the coping styles can be classified into four groups from least to most mature: narcissistic, immature, neurotic and mature. The main reason why researchers cannot scientifically classify coping styles is that it is very difficult to clarify the latent relationships among different coping styles given Classical Test Theory (CTT). In general it is hard to meet the assumptions of the test or get a true score when examining these latent relationships. It is also very difficult to separate the common factor from various specific factors or to find the nature of common factor. Additionally, relationships among various specific factors may appear to be extremely random.

Under item response theory (IRT), which can offer flexible model-based approaches to understanding response data [30], full-information item factor analysis (FIFA) has been used much more frequently in factor analysis of psychological and clinical scales [31]. The FIFA procedure marks a notable advance in the modeling of scale data and it makes use of each respondent’s complete item response vector to determine the number of underlying factors [32]. As educational and psychological constructs are typically characterized as multidimensional and lacking conditional independence, multidimensional models, such as bifactor analysis, further broaden the application of IRT in clinical, educational, and psychological assessments [33], [34]. In a bifactor model, variables are permitted to load on a general, common factor as well as on one of several specific factors [35], thus allowing a direct fit of a hierarchical model [36]. In some cases, a bifactor solution may result in empirical factors that are highly correlated, and this should clarify the relationship between factors. Such general and specific factor structures have been shown to form a credible model for various types of data involving many psychological [37] and psychiatric symptoms. These include relationships between depression and anxiety [38], [39], the structural of personality [40], [41], QOL [42] and cognitive impairment in schizophrenia [43]. In those instances, the bifactor model can create a good fit for such data. Such a model would therefore (a) provide a way to account for the general factor underlying an entire item pool in addition to the domain factors underlying item subsets [42]; and (b) account for the conditional independence of items between domains and the conditional dependence within domains [44]. This has a number of potential advantages for analyzing data systematically and for understanding related domain-specific factors independent of the general factor. First, the research can clarify relationships between domain-specific factors after extracting the general factor. For example, Xie et al. recently showed that anxiety and depression are inversely correlated after extracting the general factor of distress [39]. Second, the strength of the relationship between the domain-specific factors and their associated items can be directly examined in the bifactor model because it is reflected in factor loadings. Third, the bifactor model can be particularly useful in testing the relationship between a subset of domain-specific factors and external variables, over and above the general factor, because domain-specific factors are directly represented as independent factors [45].

Thus, the aim of this study was to adopt the full information item bifactor model to assess the dimensionality of coping styles, and to probe the latent relationship among different coping styles for stress. The present study tested the hypothesis that every item of the Coping Styles Questionnaire (CSQ) was influenced by both a general factor (coping resources) and one of six specific factors (avoidance, fantasy, self-reproach, problem solving, help seeking, and justification). We used bifactor model to model the CSQ according to the hypothesized latent factor structure. In order to support this hypothesis, a model fit comparison was conducted between one dimensional, two dimensional, six dimensional, second-order model, and bifactor model using Confirmatory Factor Analyses (CFA). If the hypothesis can be true, scores were computed on general and specific domain factors for each participant, based on IRT. Then, the scores were used to conduct the correlation analysis and to further explore relationship between other external variables (e.g., self-efficacy) and coping styles, or the relationship between different coping styles. As a result of this these analyses, this study offers relevant data and findings that clarify the specific content of coping styles. Taken together, these findings have implications for investigating patterns of coping processes and their complexities as well as providing insights into coping styles that can help maintain physical and psychological health during periods of stress.

## Methods

### Ethics Statement

The Ethics Committee of the Fourth Military Medical University specifically approved this study. All subjects were informed of the research background, purposes, and significance, and gave their written informed consent prior to participation in the study.

### Participants and Procedures

Participants in the study were military officers who took part in a 10-month military training program (N = 826). Of this, 810 valid questionnaires (mean age 23.9 years, SD = 2.4) were collected. All graduates from military academies (officers and warrant officers) have to complete these training requirements; those that perform well are eligible for early promotions and awards. Training for promotion of military skills and enhancing cohesion is conducted identically for female and male officers in a strictly closed environment with no access to outside communication. The main components of this regular military training are tasks such as completing obstacle courses and training on various types of military equipment. Daily training duration is 10 hrs, which includes marching a total of 24 km daily.

As in our previous study [46], the analysis of medical records indicated that approximately half the participants developed some training-related injuries (such as joint injuries, repetitive strain injuries, and ophthalmic infections) or serious somatic symptoms (such as stomachaches, paramenia [15.63% of females], or tarry stool [7.92% of males]) during this training. These injuries reflect the intensity and stress of the training. The total training lasted for 10 months; however, we conducted the assessments after three months of training, before any of the participants were eliminated from the program.

### Measures

#### Coping Style Questionnaire (CSQ) [24], [46]

The CSQ was administered to measure coping styles of military officer under stress. The CSQ is based on Folkman and Bond’s coping and defense questionnaire. It is a 62-item self-report test revised by Xiao Jihua and Xu Xiufeng that specifically examines Chinese language characteristics and Chinese behavioral habits (of individuals and groups) related to coping. Items are rated as 1 (agree) or 0 (disagree). The questionnaire comprises six subscales including both immature and mature coping styles. Immature coping styles include “avoidance”, “fantasy”, “self-reproach”; mature coping styles include “problem solving” “help seeking” and “justification”. In the present study, the Cronbach alpha coefficient for the CSQ was 0.847.

#### Generalized Self-Efficacy Scale (GSES) [47]

GSES was developed by Zhang and Schwarzer (1995). This is a self-report questionnaire. The Chinese version of GSES is designed to measure self-confidence in dealing with various situations that involve stress, pressure, challenges, and novel experiences. Items were rated in 4-point Likert-type scale from 1 (not true at all) to 4 (very true). The scale has shown good validity and reliability in Chinese culture. In this study, the Cronbach alpha coefficient for the GSES was 0.810.

### Data Analysis

**Figure 1:**
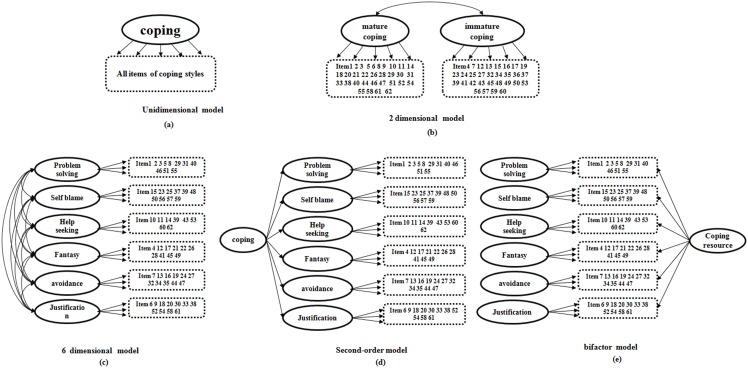
Path diagrams of 5 alternative latent variable models. Note: Model a, unidimensional model with one general factor; Model b, 2-mensional model with correlated concept factors; Model c, 6-mensional model with correlated concept factors; Model d, second-order model; Model e, bifactor model with general factor of coping resource and domain factors.

First, in order to identify the coping style model that best fits the data, a series of CFA were performed using WLSMV (Weighted Least Squares with Adjusted Mean and Variance). Four possible models of CSQ were: 1. A unidimensional, nonhierarchical two-factor model with correlated immature coping styles and mature coping styles; 2. A nonhierarchical six-factor model as the original factor structure; 3. A second-order model; 4. Hierarchical bifactor models (Figure 1). Respectively, these are one, two, and six dimensional models that are proposed on the basis of previous research [28]. Because high correlations obtain among coping styles factors, the bifactor and second-order models were also tested. As mentioned previously, the bifactor model is a hierarchical model in which every item has two factor loadings, one for a general factor, and another for one of the specific factors. In the present study, it was a hierarchical model that could be seen as a combination of the one-dimensional and six-dimensional models, and it included six domain-specific factors and one general factor.

Second, with the aim of exploring the reliability of CSQ in bifactor model, a full information item factor analysis was conducted to estimate the parameter of items such as factor loading, thresholds, and location. And the parameters of item were also used to compute the test information of CSQ. The factor scores of each participant were also computed under IRT. The full information item bifactor analysis can separate general and domain-specific factors, which are also used to estimate how much common variance is attributable to a single general factor. Therefore this type of index is able to account for common variance [48], [49]. Subsequently, in the hierarchical model, domain-specific factor loadings can be used to estimate the amount of item variance attributed to the domain factor because the general factor has been removed [31]. In turn, this provides an opportunity to further analyze the valitity of CSQ in the bifactor model.

Third, with the goal of clarifying the latent structure of coping styles including relationships between different coping styles, correlations were calculated among each of the six sub-scale scores obtained via the original six-dimensional model. In addition, the correlations of specific factor scores obtained via a bifactor analysis were also computed.

Fourth, to provide further evidence of the validity of the scale under the hierarchical bifactor analysis, the correlations between general factor and self-efficacy, as well as the correlations between self-efficacy and domain-specific (or different coping styles) before and after extracting the general factor respectively were calculated and compared. From these bifactor analyses, we could gain a more comprehensive overview of the reliability of the scale and correlations between general and specific factor scores. Additionally, correlations between specific factors and other psychological variables (self-efficacy) determined scale validity under the hierarchical bifactor analysis.

The following indices were used to evaluate the goodness of fit of the model: the Comparative Fit Index (CFI), the Root Mean Square Error of Approximation (RMSEA), and the Standardized Root Mean Square Residual (SRMR). In the current study, a model was considered to have a good fit if TLI and CFI were 0.95 or more, and SRMR and RMSEA were below 0.08.

Descriptive and correlation analyses were conducted by SPSS for Windows 16.0. Model fit comparison was conducted based on CFA completed using Mplus 7.0 [50]. Full information item bifactor analysis was conducted using POLYBIF program [36]. POLYBIF is a convenient, user-friendly operation for bifactor analysis as multidimensional IRT models. Studies have shown that this program delivers reliable results in full information item factor analysis [51].

## Results

### Overview of Model Results

A series of CFA was conducted to explore the best-fit model for CSQ. Schematics of estimated models appear in Figure 1. Fit indices of unidimensional, two-dimensional, six-dimensional, second-order, and bifactor models are shown in Table 1. Although the RSMEA and SRMR statistics indicated acceptable fits for all the tested models, for all indices the bifactor model demonstrated the best model fit. Additionally, the chi-squares of bifactor model decreased significantly when compared the other four models, which provides strong evidence of the superiority of bifactor model. Therefore, we further analyzed the reliability and validity for the bifactor model.

**Table 1:** Fit of CSQ CFA Models.

Model	df	χ^2^	CFI	RSMEA	SRMR	Δχ^2^
Unidimensional	1824	8231.64	0.87	0.066	0.069	5715.10
2-dimensional	1823	7825.59	0.89	0.067	0.067	5309.05
6-dimensional	1809	4701.91	0.91	0.045	0.054	2185.37
Second order	1794	4301.71	0.92	0.042	0.046	1785.17
bifactor	1740	2516.54	0.94	0.031	0.036	–

Note: CSQ  =  Coping Styles Questionnaire; CFA  =  confirmatory factor analysis; df  =  degrees of freedom; χ^2^ =  chi-square fit statistic; CFI  =  comparative fit index; RMSEA  =  root mean square error of approximation. △χ^2^ represent model fit comparison between unidimensional, two-dimensional, six-dimensional, and bifactor models.

### Full Information Item Bifactor Analysis of CSQ

Following an empirical comparison of the factor models, the PLOYBIF program was adopted to compute IRT parameters and factor scores for the model with the best model fit indices. As shown in Table 2, the full information item bifactor analysis demonstrated that all items loaded significantly on the general factor. This provides further evidence that the extensive variance in the diverse coping styles can be accounted for by a single general factor. Apart from general factor loading, most items also had moderate to large loadings on specific factors, which cannot be accounted for by a general factor. In this way, specific factors were separated from the general factor, and the data suggest that the specific factor, involving different coping styles, is not completely conveyed by the general factor [37]. It is worth noting that some items had negatively signed loadings on specific factors, and the most prominent items were originally grouped into the “justification” subscale in which 10 of 11 items loaded negatively on the specific factor. This may indicate that those items were negatively correlated with the original specific factor.

**Table 2:** The factor loading of CSQ.

Item	general	1 problem solving	2 self-reproach	3 help seeking	4 fantasy	5 avoidance	6 justification
1	0.654	0.318					
2	0.594	0.486					
3	0.391	0.431					
5	0.474	0.385					
8	0.186	0.374					
29	0.039	0.634					
31	0.276	0.767					
40	0.393	0.691					
46	0.278	0.595					
51	0.233	0.519					
55	0.424	0.608					
15	0.802		0.255				
23	0.526		0.416				
25	0.159		−0.174				
37	0.798		0.338				
39	0.016		−0.222				
48	0.721		0.488				
50	0.762		0.474				
56	0.721		−0.012				
57	0.728		0.501				
59	0.772		0.196				
10	0.022			0.713			
11	0.401			0.531			
14	0.375			0.283			
36	0.114			0.161			
42	0.006			0.074			
43	0.310			0.739			
53	0.274			0.603			
60	−0.041			0.748			
62	0.397			0.464			
4	0.312				0.240		
12	0.712				−0.100		
17	0.723				0.201		
21	0.624				0.096		
22	0.538				0.646		
26	0.034				0.536		
28	0.719				0.082		
41	0.395				0.205		
45	0.765				−0.028		
49	0.685				0.343		
7	0.784					0.083	
13	0.229					0.102	
16	0.641					0.112	
19	0.778					0.027	
24	0.693					0.136	
27	0.680					0.203	
32	0.436					0.030	
34	0.784					−0.046	
35	0.322					0.562	
44	0.248					0.772	
47	0.640					0.300	
6	0.383						−0.218
9	0.724						−0.118
18	0.752						−0.029
20	0.461						−0.002
30	0.62						−0.009
33	0.739						0.198
38	0.895						−0.068
52	0.274						−0.643
54	0.411						−0.760
58	0.563						−0.169
61	0.792						−0.185

**Figure 2:**
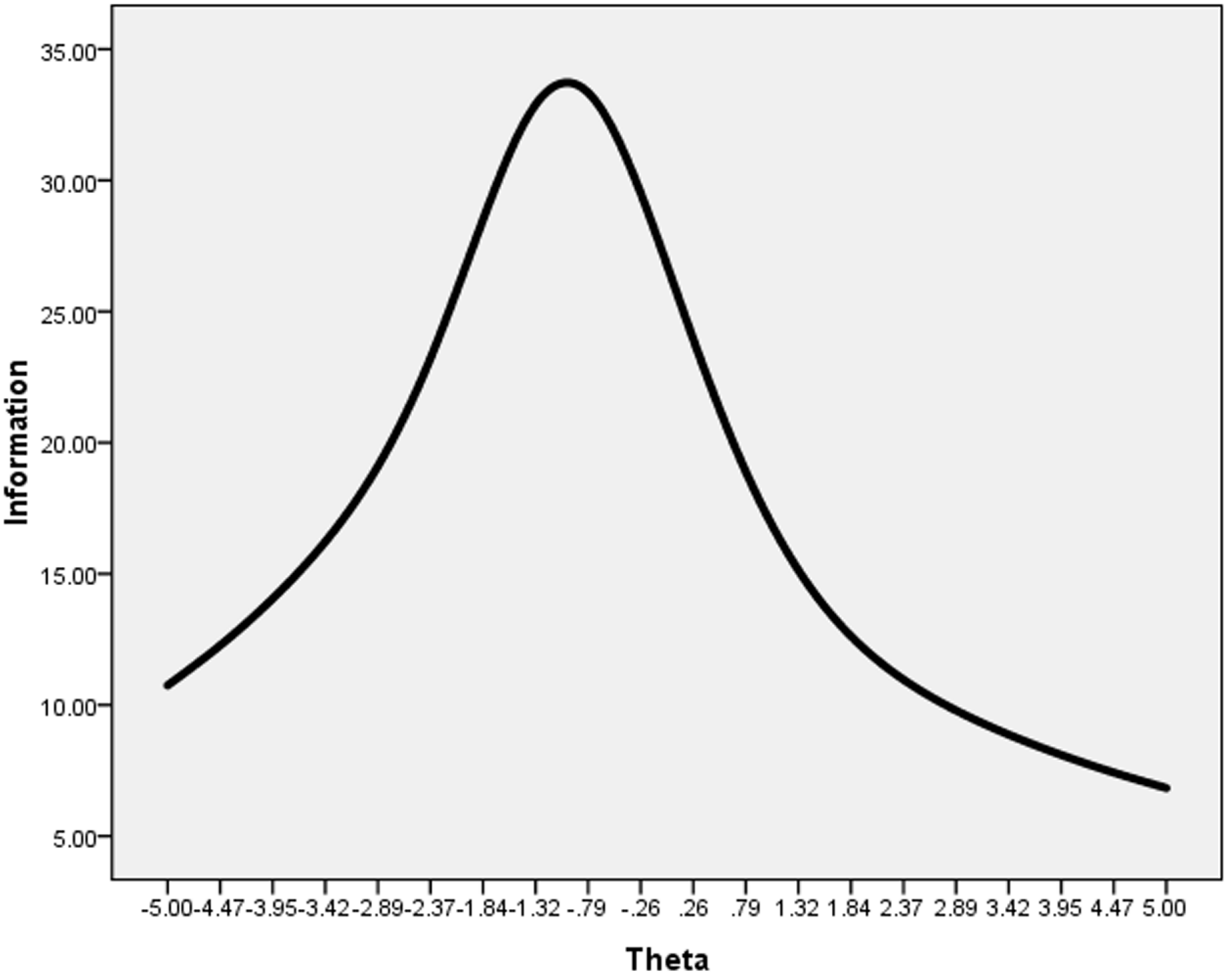
Test information of CSQ. Note: The test information curve of the CSQ based on the bifactor analysis for the general coping styles factor. X-axis represents theta of the general factor (theta), which had been standardized (0 being average, 1 being a standard deviation). The Y-axis represents the test information value. Test information is a type of reliability criterion in IRT models, the larger the test information value, the less the measurement error and the better the reliability. The test information curve was obtained by connecting all information in every theta point.

General Factor IRT parameters were used to plot test information curves for the CSQ. This can be used as a measure of reliability or it may reflect errors; the more accurate the measurement, the greater the information gained. The CSQ test information can be seen in Figure 2; this information showed that CSQ could accurately measure a general factor between (−5.0, 1.84), but was less accurate for factors larger than 2, which is to say, the interval of accuracy in this scale could be slight to moderate.

### The Relationship Among Coping Styles

For the purpose of clarifying latent relationships among different coping styles or identifying the latent construct of coping styles. We computed correlations of different coping style scores via the original six-dimensional model under CTT as well as correlations of specific factors in full information item bifactor model. In full information item factor analysis, the scores for the specific factor were separate from the general factor, and the latent correlation relationship among different coping styles can be clarified because the common factor among different coping styles is excluded. As illustrated in Table 3, which shows comparisons of correlations for the different statistical theories, changes of correlations fall into three categories.

**Table 3:** The correlation among different coping styles in CTT and bifactor analysis.

	1 problem solving(1 SF problem solving)	2 self-reproach (2 SF self-reproach)	3 help seeking(3 SF help seeking)	4 fantasy(4 SF fantasy)	5 avoidance(5 SF avoidance)	6 justification(6 SF justification)
1	1.00					
2	−.327**	1.00				
	(−.076)					
3	.242**	−.050	1.00			
	(.445**)	(-.114**)				
4	−.275**	.620**	.168**	1.00		
	(.315**)	(−.134**)	(.219**)			
5	−.296**	.630**	.147**	.705**	1.00	
	(.331**)	(−.027)	(.191**)	(.176**)		
6	−.220**	.651**	.164**	.696**	.714**	1.00
	(−.417**)	(−.004)	(−.285**)	(−.165**)	(−.355**)	

Note: the correlation coefficient in bracket was the correlation for different specific coping styles. SF problem solving  =  specific factor for problem solving, and the rest by the this analogy;

*p<0.05;

**p<0.01.

First, in one category some correlation coefficients changed either from positive into negative, or vice versa. Examples of this are correlations between problem solving, fantasy, and avoidance which showed a significant change from negative to positive when considering exclude the effect of general factors. The correlations between self-reproach and fantasy, between justification and help seeking, and between fantasy and avoidance showed a significant change from positive to negative. These correlations have undergone a qualitative change. A second category includes significant correlations that disappear after extracting the general factor. For example, significant correlations between problem solving and self-reproach, correlations between self-reproach, avoidance, and justification disappeared after extracting the general factor in full information item factory analysis. Also in this category are correlations that are not significant under CTT but become significant in IRT; an example of this is relationship between self-reproach and help-seeking. The third category contained correlations between coping styles that increased rather than decreased to some extent after the common factor was extracted. An example of this involves the relationship between coping styles of problem solving and help seeking, which strength when considering the impact of general factors.

In order to explore the relationship between general factors and other psychodynamic variables, a correlation analysis between general factor and self-efficacy was conducted. As shown in Table 4, the general factor was positively correlated with self-efficacy. The correlation between the general factor and specific factors of coping styles disappeared, which provides further evidence of the need to extract the common factor. Additionally the relationship between coping styles and self-efficacy changed; the original six-dimensional model analysis under CTT showed low to moderate significant correlations between coping styles and self-efficacy, however, the specific factor of coping styles correlation coefficient was not statistically significant under IRT except for problem solving. Specifically, the coefficient of self-efficacy and problem solving showing a positive correlation (r = 0.365, p<0.01) changed to a negative one (r = −0.260, p<0.01).

**Table 4:** The correlation between coping styles and self-efficacy.

	Self-Efficacy	problem solving(SF problem solving)	self-reproach(SF self-reproach)	help seeking(SF help seeking)	Fantasy(SF fantasy)	Avoidance(SF avoidance)	Justification(SF justification)
General factor	0.386**	0.405**	0.609**	0.251**	0.571**	0.627**	0.574**
		(0.000)	(0.154)	(0.023)	(0.045)	(0.045)	(0.140)
Self-Efficacy	-	0.365**	0.200**	−0.117*	0.148*	0.188**	0.131*
		(−0.260**)	(0.049)	(−0.019)	(0.026)	(0.019)	(0.029)

Note: the correlation coefficient in bracket was the correlation between different specific coping styles and general factor or self-efficacy;

*p<0.05;

**p<0.01.

## Discussion

The present study adopted the full information item bifactor analysis to investigate the latent relationship among different coping styles in the military officers who are under stress during military training. CFA results identified a hierarchical, bifactor model, as the best-fitting model. Full information item bifactor analysis results confirmed the existence of a general factor in different coping styles, with evidence of items loading significantly on the general factor. This hierarchical model based on IRT bifactor analysis provides an opportunity to further study the role of a general factor and latent relationship among different kinds of coping styles as well as latent relationships with other variables, such as self-efficacy. Correlation results showed that the general factor was positively correlated with coping resources such as self-efficacy. In this case, not only the correlation among different coping styles, but also the relationship between coping styles and self-efficacy, changed significantly after the general factor was excluded from overall variance. We can infer that the general factor was the main source of positive correlation between self-efficacy and coping styles, and that the latent correlation among different coping styles was concealed by the general factor.

### The Measurement of Coping Styles may be Bi-dimensional

The results of this study suggest that the measurement of coping styles is bi-dimensional, with one dimension measuring coping resources for stress and the other addressing specific coping styles. This analysis enables a clearer assessment of strategies people use in attempting to cope with stress. The general coping resource factor, underlying different coping styles, provides a reliable target for analyzing coping style management. The specific factors for coping styles may help to refine valid coping style dimensions, and assist in gaining greater understanding of defense mechanisms and health strategies related to stress. Furthermore, general and specific factors can be investigated independently in this framework. This can improve our measurement of coping styles and it may lead to more accurate results and offer a greater capacity for distinguishing among groups according to their different coping styles, a capacity we do not currently have. This bi-dimensional measurement should lead to greater conceptual clarity, and bifactor models can separate commonality shared by the facets of different styles from the unique contribution of each facet. This study also provides solid evidence for the advantages of IRT analysis in psychological assessment. In fact, the CSQ questionnaire measured coping resources and specific coping styles at the same time. While we adopted CSQ to measure coping styles for stress, two sets of scores should be reported. The first are the overall scores of coping resources, and the second are domain scores of specific scores.

### The Role of the General Factor of Coping Resources in Coping with Stress

Despite the theoretical relationship between coping resources and styles, there is a paucity of empirical data measuring the relationships between them. Perceptions of social and personal resources are integral parts of stress management. A secondary appraisal process of coping resources should be combined with a primary appraisal to determine the coping styles that individuals choose in stressful situations [8]. Hobfoll also points out that these styles and resources do not work in tandem [52], but rather they are linked to each other because selection of coping styles cannot be separated from the perception of coping resources. Therefore, the measurement of coping styles may include both the perception of coping resources and coping styles, however, it is difficult to separate them completely in CTT because of the limitations of this type of test. In this study, both CFA and full information item bifactor analysis supported the coexistence of a general factor of coping resources and specific factor structures of coping styles. Other evidence from comparisons of correlation coefficients obtained from IRT and CTT indicated that a traditional analysis showed that the positive correlation between self-efficacy and coping styles, while statistically significant, disappeared under hierarchical model. This may indicate that the general factor of coping resources is primarily responsible for link between self-efficacy and the specific factor of coping styles under the hierarchical model. Traditional first-order or second order statistical models cannot reflect these features since they cannot separate the general factor from specific factors [42]. This is the first study to provide direct evidence of the coexistence of coping resources and styles.

It is notable that some items yielded relatively high general components while others showed low components, suggesting that different kinds of coping styles may include perception of different coping resources. One example of this type of item is found on the subscale of problem solving, “able to cope with difficulties in a sensible manner.” This item had a loading of 0.802, which may indicate that respondents who picked this item felt they needed more psychological resources for coping with stress. This coincided with evidence that strengthening of specific coping resources may change and improve the coping styles. Studies on clinical patients found that people who had strong social and family support, high self-efficacy, good problem-solving skills, and positive reappraisal resources are more likely to utilize adaptive pain coping styles [53], [54]. An occupational study also verified employees who were highly resourceful made greater use of task-oriented coping strategies [55]. Other studies of normal participants also confirmed relationships between high social support resourcefulness and mature coping styles [56].

The general factor plays an important role in reflecting coping resources. This has several implications for helping people learn how to maintain mental and physical health under stressful conditions. Consistent with viewpoints in positive psychology, results for coping resources demonstrated the need of offering more psychological support and encouragement for people in stressful situations. Specifically, different specific coping resources may provide greater choices and improve the specific coping styles. On the other hand, as different coping styles may mean responding to different coping resources, the need for people to be flexible in choosing coping styles is also very important, especially when an individual may not have adequate psychological resources to cope with stress. This type of strategy may help to avoid depression and anxiety. The key to relieving stress is to adopt different coping styles according different coping resources. We also support the development of intervention models which target shared aspects of different coping styles and also tailor treatments to different coping resources for stress. These suggestions are based on results gained here by the bifactor model.

### The Latent Relationship Among Different Coping Styles

Comparisons of correlations among coping styles taken before extracting the general factor with those computed after extracting this general factor revealed significant differences. This outcome is largely because due to the fact that different coping styles share substantial variances that are accounted for only by the single general factor of coping styles. Traditional psychometric theory can reflect observed variables features but is not able to account for the latent variable mechanisms here [32]. For example, the correlations between problem solving, fantasy, and avoidance may be negative in CTT, and this would seem reasonable on the basis of psychodynamic theory. Problem solving motivates action, and may sometimes initiative to deal with stress situation effectively. Fantasy and avoidance, on the other hand, feature behavioral inhibition, and tend to undermine action, seems to have no benefit in stressful conditions. However, this correlation between them was positive under full information bifactor analysis, although problem solving and avoidance or fantasy may indicate that people have different resources, but uses the same coping strategy when they face of stressful situations [54]. That is people who have problem solving strategies adopt more coping resources, but all the these strategies were aimed at reducing the psychological burden of stress. Additionally, the general factor of coping styles may obscure the latent relationships between different coping styles. Problem solving, avoidance, and fantasy may belong to the same clusters of defense mechanisms since they showed positive correlations with each other. Studies have shown that the same clusters of defense mechanisms appear to be correlated with independent and objective measures of mental health [57]; this may have implications for enriching and developing theories of psychological treatment, as well as improving and supplementing the content of health behaviors in different stressful situations. Immature coping styles such as fantasy or avoidance may be effective ways to cope in order to maintain mental health when people with poor coping resources find themselves in high-pressure situations. Another extreme example differs from results from CTT; this involves a relationship between help seeking and justification which shows significant negative correlation rather than significant positive correlation. This may indicate different clusters of defense mechanisms; therefore it is possibly an oversimplification to attribute these clusters to the mature coping styles because the defense mechanisms underlining help seeking and justification may be inconsistent. Those results also have implications for maintaining health under stress. There is no need to stick to one or another category of coping style because so-called mature or immature coping styles may have the same coping mechanism for dealing with stress. Some kinds of coping styles (e.g. problem solving and avoidance) may have similar implications for health under stressful conditions.

Our study has limitations. Firstly, it is a cross-sectional study. Although we provide direct evidence of the general factor of coping resources and have discovered the latent relationship among different kinds of coping styles, it is difficult to establish causal relationships between coping styles and health in stress. An in-depth study should be conducted to study the latent mechanisms of different coping styles and the relationships between specific coping styles on mental health. Future longitudinal or experimental studies will facilitate requisite causal evaluations on these relationships. Second, this research provides an example of how the advanced techniques of IRT can be applied to explore the latent relationship among complex psychological mechanisms and reveal a more detailed internal relationship among the variables. However, the central interest in the bifactor model involves the effect of the general factor; domain-specific factors are secondary [42]. Therefore, the analysis of the relationship between the specific factor and external criteria may not be adequate. Finally, the stress condition here is quite distinct, as it is confined to a military situation, and its generality under different kinds of stress should be studied in the future.
